# Plate fixation of small metacarpal and metatarsal bone fractures in 27 horses

**DOI:** 10.1111/vsu.70032

**Published:** 2025-10-02

**Authors:** Virginia Melly, Kyla F. Ortved, Holly L. Stewart, Darko Stefanovski, Dean W. Richardson, Kirstin A. Bubeck, Patricia M. Hogan, José M. García‐López

**Affiliations:** ^1^ Department of Large Animal Clinical Sciences College of Veterinary Medicine, Michigan State University East Lansing Michigan USA; ^2^ Department of Clinical Studies‐New Bolton Center University of Pennsylvania Kennett Square Pennsylvania USA; ^3^ Department of Clinical Sciences Cummings School of Veterinary Medicine, Tufts University North Grafton Massachusetts USA; ^4^ Hogan Equine at FairWinds Farm Cream Ridge New Jersey USA

## Abstract

**Objective:**

To present fracture cases selected for plate fixation (PF), describe surgical techniques and assess their association with postoperative complications and return‐to‐work outcomes in horses undergoing PF for small metacarpal/metatarsal (SMCT) fractures.

**Study design:**

Multicenter retrospective study.

**Animals:**

A total of 27 horses.

**Methods:**

Horses treated with PF of a SMCT fracture between 2008 and 2023 across three hospitals were included. Medical records and imaging were reviewed. Long‐term outcomes were established by readmission to the hospital, telephone interviews, and/or race records. Univariable and multivariable regression models evaluated variables associated with return to intended use.

**Results:**

PF of fractures was performed in 27 horses. Fractures were all in the proximal SMCT, with 20/27 (74.1%) articular, 22/27 (96.3%) comminuted, and 24/27 (81.5%) displaced. Of horses with follow‐up, 19/25 (76%) returned to prior work level. Postoperative complications occurred in 9/27 (33.33%) horses. No individual explanatory variable was significantly associated with return to use in the univariable analysis. Racehorse earnings per start were lower postoperatively compared to preoperatively (*p* = .02).

**Conclusion:**

Most horses treated for SMCT fractures with PF returned to prior work level, although postoperative complications were common. None of the explanatory variables were significantly associated with return to use.

**Clinical significance:**

PF for proximal SMCT fractures is effective, yielding high return‐to‐work rates and better outcomes than previously reported case series.

## 1 INTRODUCTION

Small metacarpal and metatarsal bone (SMCT; metacarpals/metatarsals 2 and 4) fractures are common and typically result from external trauma or overload injury.^1^ Treatment, including conservative or surgical management, depends on factors such as fracture location, chronicity, instability, and whether the fracture is open or closed.^2^ Surgical options include distal fragment removal, segmental ostectomy, complete removal of the entire fourth metatarsal bone, fracture debridement, or internal fixation.^3^, ^4^, ^5^, ^6^ Internal fixation, using screws and/or plates, is preferred for proximal fractures requiring axial stability.^5^ SMCT in horses contribute to carpometacarpal and tarsometatarsal joint stability in a compressive and torsional loading environment.^7^ SMCTs function as lever arms, rotating at their proximal attachments, where the carpal or tarsal collateral ligaments exert the most significant tensile forces. The interosseous ligament counterbalances these rotational forces to preserve proper orientation. Removing part of the bone and ligament shortens the lever arm, which increases the risk of luxation and rotation.^5^

Complications are reported to occur following SMCT fracture treatment (14%–33%^2^, ^8^), and include instability, excessive callus formation, non‐union, third metacarpal/metatarsal (MC3/MT3) fractures, osteomyelitis, lameness, osteoarthritis of the carpometacarpal/tarsometatarsal joints, and suspensory desmitis.^2^, ^8^ Many of these complications result from persistent instability due to altered lever arm function, secondary to a reduction in its effective length. Internal fixation restores joint alignment and preserves the splint bone's integrity, ensuring even force distribution and minimizing stress at the fracture site.^5^ It functions as a tension band, converting the tensile forces exerted by the collateral ligaments into compressive forces across the fracture site. This technique can potentially reduce complications and decrease the likelihood of excessive callus formation in SMCT fractures.

Internal fixation for SMCT fractures was first reported in 21 horses finding that screw fixation alone had a high failure rate, whereas plate fixation (PF) of 11 horses yielded better outcomes, with 54.5% of horses returning to their previous level of work.^5^ Two retrospective studies, involving five and seven horses, respectively, reported variable return‐to‐work rates (33%–60%) and a prolonged convalescence period averaging 6.5 months.^2^, ^8^ Sherlock et al. focused solely on open fourth metatarsal fractures, while Jackson et al. included both open and closed fractures.

Both studies were limited by small sample sizes, short follow‐up durations, and lack of focused evaluation of PF efficacy, limiting their prognostic value.

There remains a paucity of literature specifically examining PF for SMCT fractures. The objective of this study was to present cases selected for PF, describe surgical techniques, and evaluate factors that may affect return‐to‐work outcomes in horses undergoing SMCT PF. We hypothesized that PF would result in a higher rate of return‐to‐work compared to previous studies in which PF of SMCT fractures was described. The outcomes of this study were intended to improve the current understanding of SMCT fractures and their impact on prognosis.

## 2 MATERIALS AND METHODS

### 2.1 Animals

Medical records and diagnostic imaging from three referral hospitals (January 2008–November 2023) were reviewed for horses with SMCT fractures treated with PF. Inclusion criteria required SMCT fractures managed with PF, available pre‐ and postoperative radiographs and a minimum of 10 months of follow up information. Cases treated with screws alone or lacking complete imaging were excluded. Information obtained from the medical records included patient signalment (age, breed, sex, discipline), affected SMCT, history, degree of lameness (AAEP scale^9^), fracture classification, presence of comorbidities (yes/no), surgical details, antimicrobial administration, postoperative care, complications (intra‐ and postoperative), and short‐ and long‐term outcomes, including return to use (yes/no), return to previous level of work or performance (yes/no), and time to return (see *Methods: Outcome* for details).

### 2.2 Imaging analysis and surgery

All available radiographic and computed tomography (CT) imaging was reviewed by a board‐certified large animal equine surgeon and a large animal surgery resident. Preoperative imaging was assessed for fracture classification; location (proximal third, middle third, or distal third of the bone), presence of displacement, presence of comminution, articular involvement (yes/no), presence of callus (yes/no), and fracture configuration (simple oblique, sequestrum). PF and implant selection were not guided by a standardized protocol but were influenced by individual surgeon preference and clinical assessment of fracture characteristics, including factors such as configuration, comminution, displacement, and fracture status (open vs. closed). The implants used, MC3/MT3 engagement, bone graft use, additional procedures performed, and intraoperative complications were recorded. Perioperative antimicrobials were administered based on surgeon preference and fracture status (open vs. closed). Comorbidities, including suspensory desmitis, carpal or tarsal bone fractures/fragments, or other conditions which might have an effect on outcome (Table 1), were noted. Postoperative care was surgeon‐determined.

**TABLE 1:** Signalment, discipline, limb and bone affected, fracture configuration, implant details, relevant surgical information and comorbidities, complications, and return to use of each horse.

Horse	Signalment	Discipline	Limb	Bone	Fracture description	Plate used	Screws used	Surgical information and comorbidities	Complications	Plate removal	Return
1	13 yo WB M	Dressage	RH	MT4	P, Co, D	6 hole 3.5 mm recon	3.5 mm cortical	Bone graft	N	N	Y
2	2 yo TB F	Racing	LF	MC2	P, A, Co, D	6 hole 3.5 mm LC‐DCP	3.5 mm cortical		N	N	Y
3	9 yo TB M	Hunter	LF	MC4	*P, A, Co, D	6 hole 3.5 mm recon	3.5 mm cortical	Bone graft	Proximal fragment displacement	Y	Y
4	9 yo WB G	Hunter	RH	MT4	P, A, Co, non D	8 hole 3.5 mm Na LCP	3.5 mm locking 3.5 mm cortical 2.7 mm cortical		N	N	Y
5	4 yo TB S	Racing	LF	MC2	P, A, Co, D	5 hole 3.5 mm recon	3.5 mm cortical	Incomplete chronic C3 slab fracture, First screw engaging MC3	Incomplete fracture healing, progressive CMC OA	Y	Y
6	2 yo TB F	Racing	RF	MC2	P, A, Co, non D	4 hole 3.5 mm recon	3.5 mm cortical	Carpal osseous fragments	N	N	N
7	5 yo TB G	Racing	RF	MC4	P, A, Co, D	7 hole 3.5 mm recon	3.5 mm cortical	Minimally invasive, all engage MC3	Proximal fragment displacement, CMC OA	Y	Y
8	4 yo TB G	Racing	LF	MC2	P, A, Co, D	7 hole 3.5 mm Na LC‐DCP	3.5 mm cortical		N	N	Y
9	9 yo TB G	Pleasure	RH	MT4	P, A, Co, D	5 hole 3.5 mm recon	3.5 mm cortical	T4 fracture	N	N	Y
10	3 yo TB C	Racing	LF	MC2	P, Co, D	5 hole 3.5 mm Na LC‐DCP	3.5 mm cortical	Proximal MC3 fragment, castration	N	N	Y
11	2 yo TB C	Racing	RF	MC2	P, A, Co, D	5 hole 3.5 mm Na LC‐DCP	3.5 mm cortical	C3 fragment	N	N	Y
12	18 yo TB X M	Eventer	LH	MT4	P, Co, D	10 hole 3.5 mm recon	3.5 mm cortical		N	N	Y
13	9 yo TB G	Eventer	LH	MT4	P, Co, non D, Ca	8 hole 3.5 mm recon	3.5 mm cortical		N	N	Y
14	2 yo TB C	Racing	RF	MC2	P, A, Co, D	6 hole 3.5 mm Na LC‐DCP	3.5 mm cortical		N	N	Y
15	18 yo TB G	Hunter	LH	MT4	*P, A, Co, D	9 hole 3.5 mm recon	3.5 mm cortical		Wound dehiscence, TMT OA	N	N
16	3 yo TB G	Racing	RF	MC2	P, A, Co, D	5 hole 3.5 mm recon	3.5 mm 4.5 mm cortical	Distal radial and intermediate carpal fragments	Stripped threads	N	Y
17	2 yo TB G	Racing	LF	MC2	P, A, Co, D	5 hole 3.5 mm Na LC‐DCP	3.5 mm cortical		N	N	N
18	3 yo TB G	Racing	RF	MC2	P, A, Co, D	6 hole 3.5 mm Na LC‐DCP	3.5 mm cortical			N	N
19	18 yo WB G	Jumper	LH	MT4	P, A, Co, D	10 hole 3.5 mm recon	3.5 mm cortical and 3.5 mm cancellous		Stripped threads	N	N
20	4 yo WB G	Jumper	RH	MT4	P, Co, non D, Ca	8 hole 3.5 mm recon	3.5 mm cortical	Previous sequestrectomy, SD, collagen sponge	N	N	Y
21	11 yo Fjord G	Therapy/lesson	LF	MC4	*P, A, Co, D	8 hole 3.5 mm recon	3.5 mm cortical	3 screws engage MC3	Displaced fragment, SSI, laminitis	N	N
22	7 yo WB G	Eventer	LF	MC4	P, Co, non D, Seq	6 hole 3.5 mm recon	3.5 mm cortical, 3.5 mm BSF	Sequestrectomy, bone graft	Stripped threads	N	N
23	2 yo TB F	Racing	LF	MC2	P, O, A, D	6 hole 3.5 mm recon	3.5 mm cortical, 3.5 mm BSF		Stripped threads, screw loosening, excessive callus formation	N	Y
24	2 yo TB G	Racing	LF	MC2	P, A, Co, D	5 hole Na LC‐DCP	3.5 mm cortical		N	N	Y
25	16 yo TB M	Pleasure	LH	MT4	*P, A, Co, D	8 hole 3.5 mm Na LCP	3.5 mm locking	Proximal MT3 fracture repaired, minimally invasively	SSI, TMT OA, MT3 screw pain	N	Y
26	13 yo TB M	Dressage	LF	MC4	*P, A, Co, D	7 hole 3.5 mm Na LCP	3.5 mm cortical, 3.5 mm locking		Wound dehiscence, CMC OA	N	Y
27	6 yo QH G	Showing	LH	MT4	P, Co, D	7 hole, 3.5 mm LCP reconstruction plate	2 3.5 mm cortical, 3.5 mm locking		MT3 catastrophic fracture	N	N

Abbreviations: A, articular; BSF, bone screw fastener; C, colt; C3, third carpal bone; Ca, callus; CMC, carpometacarpal joint; Co, comminuted; D, displaced; Ex, exostosis; F, filly; G, gelding; LC‐DCP, limited contact dynamic compression plate; LCP, locking compression plate; LF, left front; LH, left hind; M, mare; MC3, third metacarpal; MT3, third metatarsal; N, No; Na, narrow; O, oblique; P, proximal third; QH, Quarter Horse; Recon, reconstruction plate; RF, right front; RH, right hind; S, stallion; SD, suspensory desmitis; S, sequestrum; SSI, surgical site infection; T4, fourth tarsal bone; TB, Thoroughbred; TMT, tarsometatarsal joint; WB, Warmblood; Y, yes; yo, year old. * Open fracture.

### 2.3 Outcome

Follow‐up information, a minimum of 10 months postoperatively, included return to work, work level, time to return to ridden exercise, complications, and available imaging. Sources included clinical and competition records, and informal phone communication with the referring veterinarian, owner, or trainer. Racing performance data collected from Equibase (www.equibase.com) included earnings per start, number of starts, and placings pre‐ and post‐surgery. Time to first race post‐surgery was calculated from surgery date to race date. Plate removal or additional associated surgeries following PF were recorded.

### 2.4 Statistical analysis

A single investigator organized data in Microsoft Excel for analysis. Categorical variables are reported as frequency counts (percentages), and continuous variables were assessed for normality using the Shapiro–Wilk test. Parametric data are reported as mean ± SD, and non‐parametric data as median and range. Univariable logistic regression assessed associations between explanatory variables and return to use for all data and return to the previous level in non‐racehorses. Explanatory variables assessed were: sex, age, breed, athletic use, affected limb, affected bone, laterality (left vs. right), end (hind vs. front), fracture status (open vs. closed), plate type, plate hole numbers, comorbidities, surgeon, and postoperative complications. In racehorses, a Wilcoxon signed‐rank test was used to compare pre‐ and postoperative starts, earnings, earnings per start, and placings. Univariable logistic regression analyzed plate type and postoperative earnings in relation to complications. Mixed effects logistic regression included horse as a random effect. Independent variables from univariable analysis with *p* < .2 were retained in multivariable logistic regression model, with model fit assessed using Akaike information criterion (AIC). The final model used stepwise backward selection to remove non‐significant variables, with significance set at *p* < .05. For multivariable models, data was assessed as a complete data set, and as smaller data sets by use (i.e., racehorse vs. non‐racehorse). The *χ* ^2^ test was used to assess if there was a difference in return to use for racehorses versus non‐racehorses. Multivariable models are presented as odds ratios (ORs) and 95% CIs. Data were analyzed in R (version 4.2.2) using RStudio (version 2022.12.0 + 353 using base R package, dplyr, MASS, lme4, and logistf ).

## 3 RESULTS

### 3.1 Signalment and clinical findings

Table 1 summarizes clinical findings for 27 horses treated with SMCT PF. Thoroughbreds were the most common breed (20/27, 74.1%), and geldings were the most prevalent sex (13/27, 48.1%). The median age was 5 years (range: 2–18). Racing was the most common discipline (13/27, 48.1%), followed by show hunting (5/27, 18.5%).

Lameness grade was recorded in 8/27 horses, with a median grade of 3.5/5 (range: 0–4).^9^ Data on the chronicity of the fracture prior to PF were available for seven horses, with a median of 10.5 weeks (range: 1–80 weeks).

Forelimb SMCT fractures (22/27, 81.5%) were more common, with the left fore second metacarpal (MC2) bone most frequently affected (10/27, 37%), followed by the left hind MT4 (8/27, 29.6%).

Table 1 outlines fracture descriptions. Radiographs served as the primary tool for characterizing fractures. Preoperative CT was used in seven cases to refine fracture characterization, identify additional injuries, and aid surgical planning. In Horse 9, CT revealed an unrecognized concurrent fourth tarsal bone fracture. All 27 fractures were located in the proximal third of the SMCT. A total of 20 of 27 (74.1%) were articular, 26/27 (96.3%) were comminuted, 22/27 (81.5%) were displaced, 1/27 (3.7%) was simple oblique and 5/27 (18.5%) were open. One fracture (Horse 20) had callus, and one (Horse 22) was diagnosed with a sequestrum. Preoperative radiographs diagnosed carpometacarpal osteoarthritis due to a chronic, incomplete C3 fracture in Horse 5.

Horse 20 was previously treated for an open comminuted proximal right hind MT4 fracture with sequestrectomy 4 months earlier, presented with a transverse, non‐displaced fracture within the exuberant callus from the prior sequestrum removal.

Suspensory desmitis due to axial impingement of the affected SMCT was confirmed via ultrasonography in only one horse (Horse 20) and adhesions between the suspensory ligament and the SMCT were present and resected intraoperatively. Comorbidities, listed in Table 1, were present in 7/27 (25.9%) horses.

### 3.2 Surgery and postoperative care

All horses were placed in lateral recumbency under general anesthesia with the affected SMCT up. A tourniquet was applied for hemostasis as needed. Contaminated and necrotic tissues were debrided, and bone fragments and osseous callus were removed. Fluoroscopic or radiographic guidance was used for plate placement.

**FIGURE 1:**
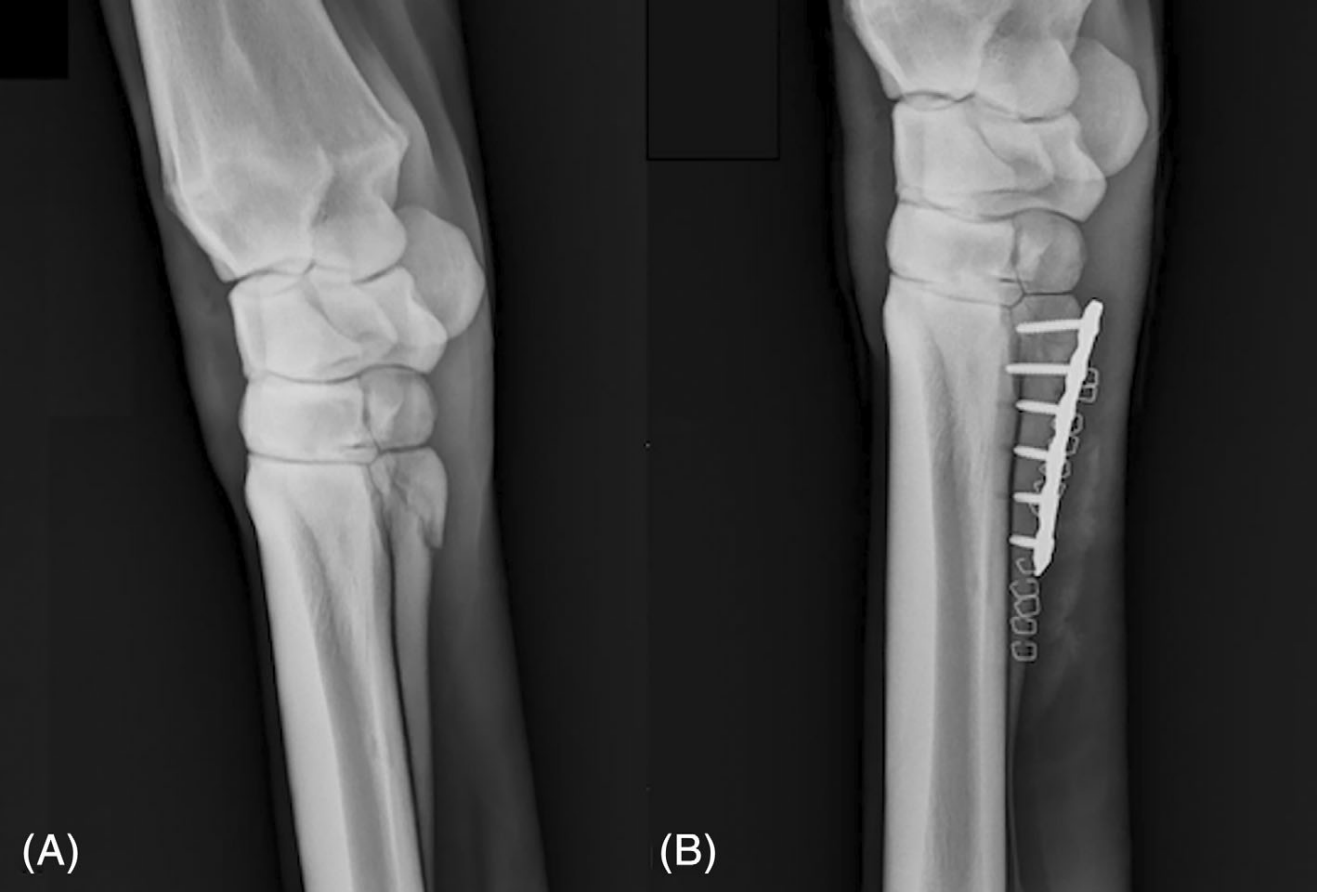
(A) Preoperative radiograph of proximal articular comminuted and displaced left fore second metacarpal fracture in Horse 2. (B) Postoperative radiograph after repair with a six‐hole 3.5 mm limited contact‐dynamic compression plate and 3.5 mm cortex screws. Note evidence of drilling into the plantar cortex of third metacarpal (MC3).

**FIGURE 2:**
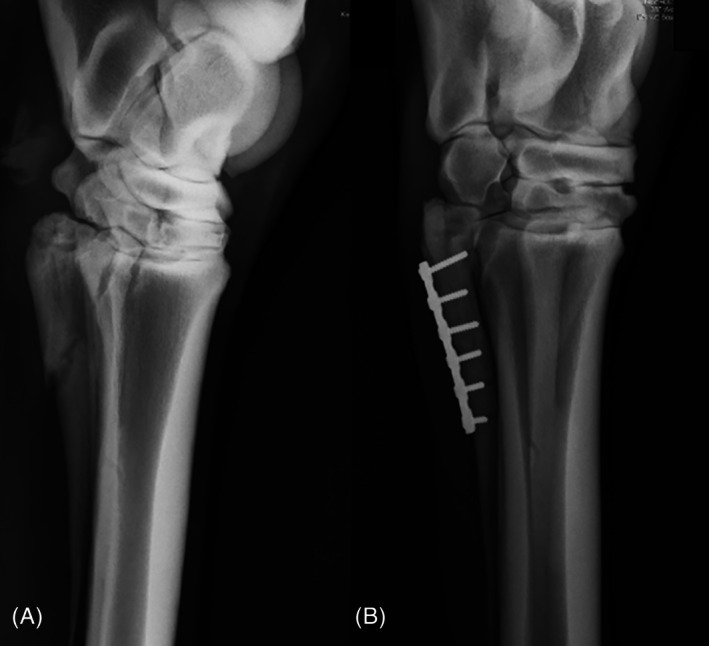
(A) Preoperative radiograph of proximal comminuted and displaced right hind fourth metatarsal fracture in Horse 1. (B) Postoperative radiograph after repair with a six‐hole 3.5 mm reconstruction plate and 3.5 mm cortex screws.

**FIGURE 3:**
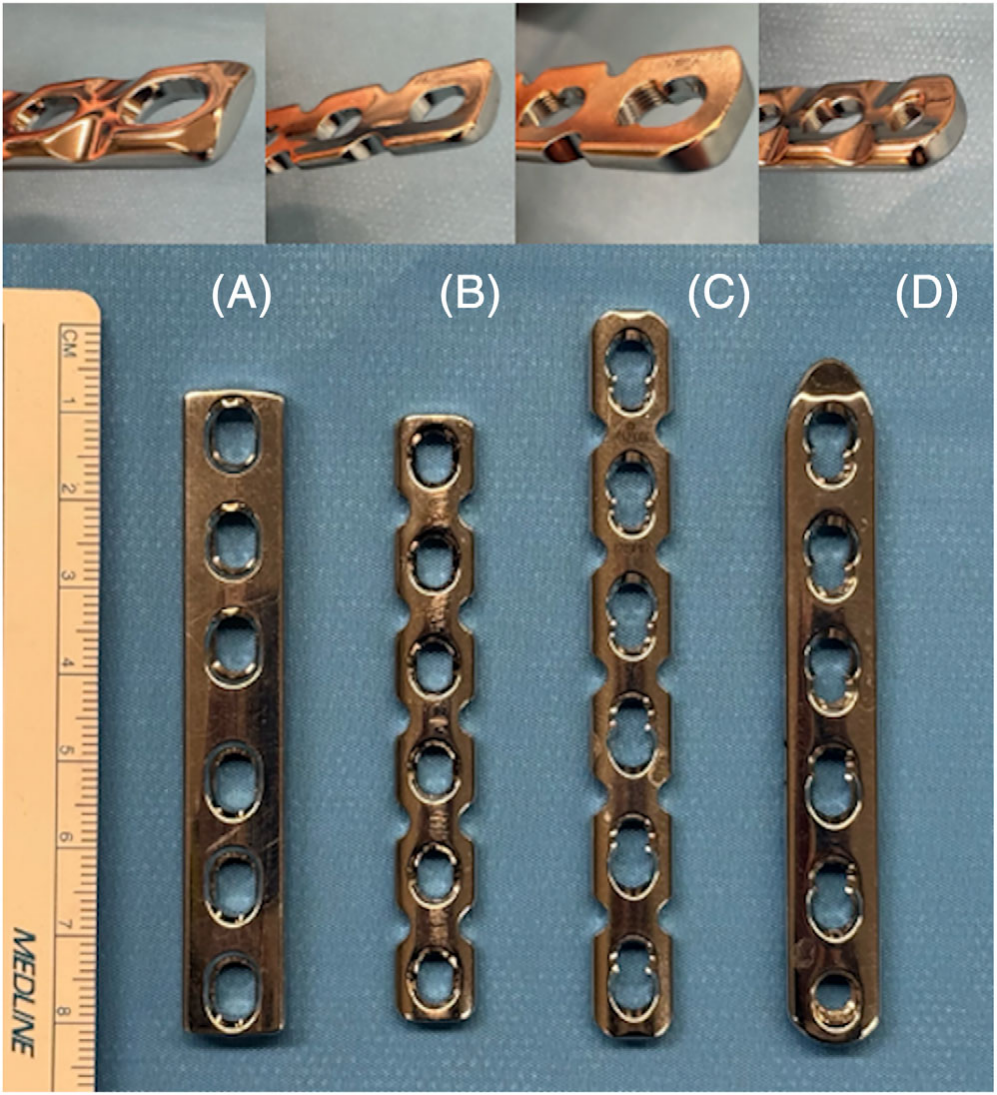
Comparison of plate appearance and their measurements used for repair (facing forward [bottom] and oblique underside [top]). All plates contain six holes. (A) 3.5 mm limited contact dynamic compression plate‐thickness 3.3 mm, width 11 mm, hole spacing 13 mm. (B) 3.5 mm reconstruction plate‐thickness 2.8 mm, width 10 mm, hole spacing 12 mm. (C) 3.5 mm locking compression plates (LCP) reconstruction‐thickness 3.5 mm, width 10.1 mm, distance between center of holes 14.0 mm, distance between central holes 10 mm. (D) 3.5 mm LCP‐thickness 3.4 mm, width 11 mm, distance between center of holes 13, distance between central holes 9.4 mm.

Plate types included limited contact‐dynamic compression plates (LC‐DCP, 8/27, Figure 1), locking compression plates (LCP, 2/27), and reconstruction plates (16/27, Figure 2), all of which were 3.5 mm (Figure 3). One reconstruction plate was a locking type. When an LCP was used, a 3.5 mm narrow plate was chosen. Screw types included locking head screws, cortex screws, and bone fasteners (OsteoCentric Technologies Austin, Texas). In Horses 22 and 23, three self‐tapping fasteners were used due to stripped threads from prior cortical holes. Horse 4 had a 2.7 mm cortex screw placed in lag fashion. Horse 19 had a 3.5 mm partially threaded cancellous screw placed after a 3.5 mm cortex screw stripped. Conventional 3.5 mm or 4.5 mm cortical or locking head screws were used in all other cases.

Screws engaged MC3 in three cases for added stabilization due to small fragment size or degree of comminution. Autologous cancellous bone grafts from the tuber coxae were used in three horses (Table 1) to fill fracture gaps in cases of chronic non‐union fractures. In Horse 20 with suspensory desmitis, an amikacin‐infused collagen sponge (1.25 g) was placed between the affected SMCT and the suspensory ligament to prevent adhesion formation. Two fractures were repaired minimally invasively, using plate tunneling and stab incisions for screw placement.

Complete records of antimicrobial administration were available in 19/27 cases. The standard regimen, used in 11 cases, consisted of potassium penicillin (22 000 IU/kg IV every 6 h) and gentamicin sulfate (8.8 mg/kg IV every 24 h), given preoperatively and continued for 1–3 days postoperatively. In three cases, including one open fracture, a 3‐to‐10‐day course of oral trimethoprim‐sulfamethoxazole (30 mg/kg orally every 12 h) was prescribed following administration of potassium penicillin and gentamicin. For three open fractures, antimicrobial treatment was extended pre‐ and postoperatively. Local antimicrobial strategies using amikacin or ceftiofur, such as intravenous regional limb perfusions (2.5 g amikacin or 1 g ceftiofur), impregnated calcium sulfate beads (Kerrier, WestPalm Beach, Florida) or collagen sponges (Ultrafoam, Davol Inc., Warwick, Rhode Island) were recorded in 13 cases. The median number of regional limb perfusions performed during the perioperative period was 2 (range: 1–7 times). The mean surgery time was 88.26 min (SD ±19.63 min). Intraoperative complications included thread stripping in four horses. The median postoperative hospitalization time was 2 days (range: 1–34 days). Postoperative recommendations included strict stall rest for a median of 4 weeks (range: 2–10 weeks), followed by a gradual hand‐walking program for a median of 4 weeks (range: 0–12 weeks). A recheck evaluation, including radiographs, was recommended at a median of 8 weeks (range: 4–12 weeks). If healing was appropriate, small paddock turnout was advised for a median of 4 weeks (range: 3–6 weeks) before resuming ridden exercise. The estimated return to training was 3 months for all racehorses repaired with an LC‐DCP and 6 months (range: 6–7 months) for all other horses.

Plate removal was performed in three horses at a mean of 4.67 months postoperatively (SD ± 3.055). Of these three horses, two horses (66.7%) had screws engaging the third metacarpal/metatarsal bone (MC3/MT3). Plate removal was performed under general anesthesia in all horses. In one case, the plate was removed due to incomplete fracture healing (Horse 5, 8 months postoperative). In others, removal was elected based on surgeon recommendation (Horse 7), or owner request (Horse 19). The screws repairing the additional MT3 fracture in Horse 30 were removed 4 months postoperatively.

### 3.3 Postoperative complications

**FIGURE 4:**
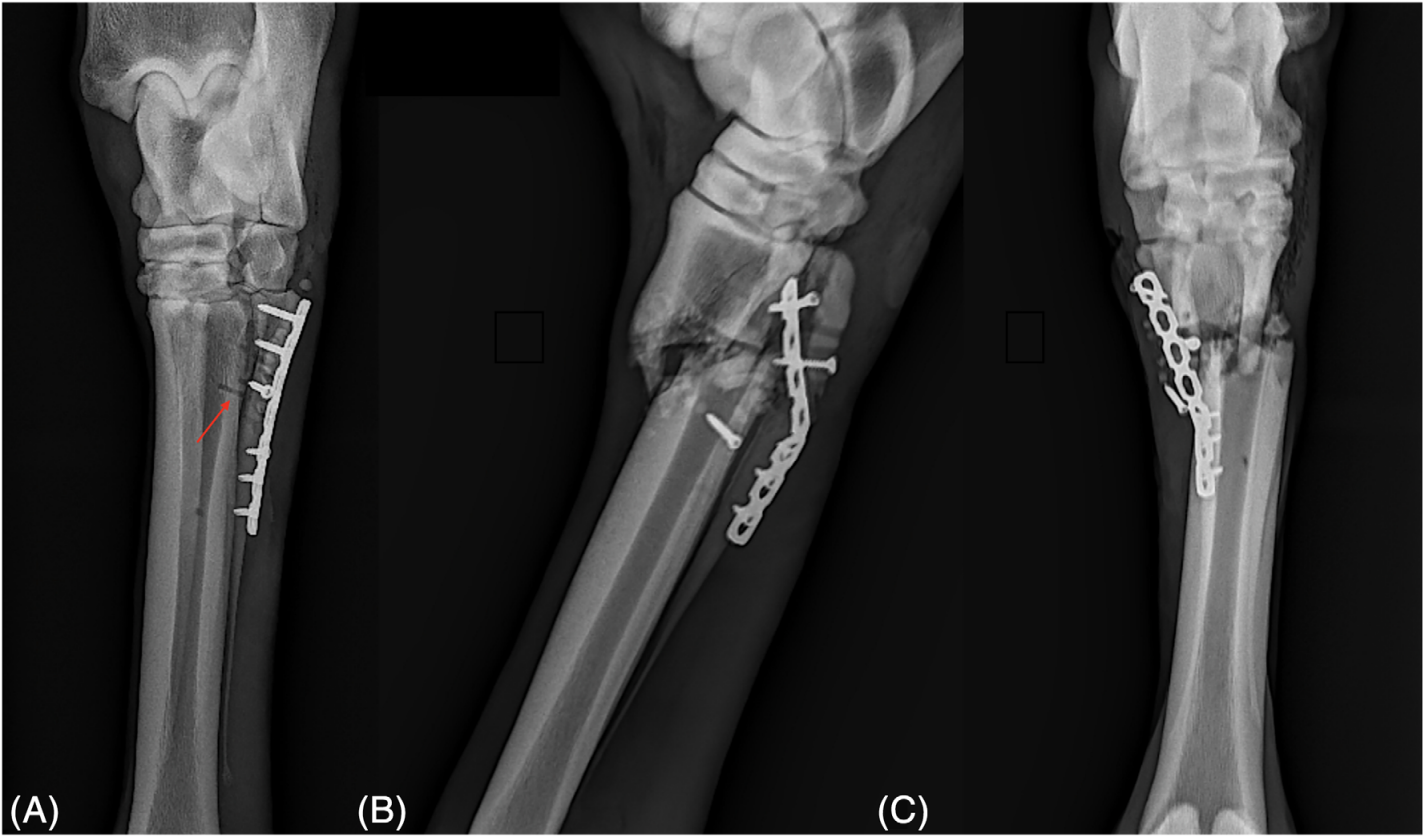
(A) Postoperative radiograph of repair of a proximal comminuted and displaced left hind fourth metatarsal fracture with a seven‐hole 3.5 mm locking compression reconstruction plate. Red arrow highlighting drill hole in third metatarsal (MT3) plantar near site of MT3 fracture location. (B, C) Postanesthetic recovery radiographs demonstrating a complete left hind proximal third metatarsal bone fracture.

Postoperative complications occurred in 9 of 27 horses (33.3%), with a total of 16 individual complications recorded (Table 1). Horse 27 sustained a catastrophic MT3 fracture in the same limb that had previously undergone MT4 repair with plate fixation, during anesthetic recovery, and was subsequently euthanized (Figure 4). Horse 21 developed support limb laminitis due to a surgical site infection and was also euthanized. Other complications included wound dehiscence (2 horses), surgical site infection (2 horses), incomplete fracture healing (2 horses), excessive callus formation (1 horse), tarsometatarsal/carpometacarpal osteoarthritis (5 horses), screw loosening (1 horse) and postoperative fracture displacement (3 horses).

### 3.4 Outcome

Of the 27 horses, 23 had a minimum of 10 months follow‐up (range: 10–180 months). Two horses were lost to follow up and two were euthanized postoperatively as described below. Overall, 19 of the 25 horses (76%), with available follow up, returned to their previous level of work or higher, including nine of 14 non‐racehorses (64.3%) and 10 of 11 racehorses (90.9%).

The mean time to return to ridden exercise for non‐racehorses was 6.5 months (SD ±1.916), while the mean time to return to racing for racehorses was 8.94 months (SD ±5.16). All racehorses raced at least twice before surgery. Two non‐racehorses returned to a lower work level. Two horses were retired: Horse 22 due to cervical osteoarthritis and ataxia and Horse 6 as a broodmare. Two horses were euthanized from complications, including support limb laminitis and a catastrophic MT3 fracture during recovery. Postoperative imaging (10/27 horses) revealed recorded complications, while others showed appropriate healing or recovery.

### 3.5 Statistical analysis

No association between explanatory variables and return to use was identified in univariable regression analysis. All variables were included for backward multivariable model selection; however, significant variables were not identified. Analysis of race records for the 10 racehorses that returned to work showed no differences in pre‐ and postoperative starts (*p* = .202), total career earnings (*p* = .625), or race placings (*p* = .09). Median earnings per start were lower postoperatively compared to preoperatively (*p* = .02). Preoperative median earnings per start were $5199 ($718–$40 833 range), while postoperative median earnings per start were $2735.20 ($372.10–$7176.10 range).

## 4 DISCUSSION

Our study reports a 76% success rate in horses with SCMT fractures repaired by PF, where success is defined as a return to the previous level of work or higher. Among racehorses, 10 of 11 (90.9%) returned to racing, while nine of 14 (64.3%) non‐racehorses returned to their previous level of work. Although the proportion was higher in racehorses, no difference was detected (*p* = .93). Earnings per start in racehorses were lower after surgery compared to before (*p* = .02). No other factors evaluated affected return‐to‐work outcomes in these horses undergoing SMCT PF. These findings suggest a higher success rate and more favorable outcomes in this study population compared to previously reported studies of SMCT PF.^2^, ^5^, ^8^

Thoroughbred racehorses comprised 74.1% of our cohort, and all fractures in this group involved the medial splint bone (MC2) and were closed. This pattern suggests a predominance of overload or stress‐related injury mechanisms, rather than acute external trauma, although racing‐related traumatic events cannot be entirely excluded. The anatomical role of MC2 in supporting the second carpal bone likely contributes to its higher susceptibility to overload fractures compared to other splint bones.^10^ Previous studies report MT4 as the most affected bone in proximal SMCT fractures, while our study findings of a predominance of left MC2 fractures likely reflect the unique biomechanical stresses experienced by Thoroughbred racehorses in our population.^2^, ^4^, ^8^, ^11^ The high proportion of racehorses in our cohort explains this difference and underscores the influence of population characteristics on fracture patterns associated with repetitive high‐speed loading.

Proximal SMCT fractures made up all of the cases aligning with the commonly recommended approach of internal fixation for managing these fractures, especially when they are closed and non‐comminuted.^1^, ^5^, ^12^ Only five fractures in our study were open with external trauma confirmed in just three cases, contrasting with previous literature that reports a higher prevalence of open fractures (43%–62%) and external trauma (66%–100%) in proximal SMCT fractures.^2^, ^5^, ^8^ While previous studies have reported acute fractures (<3 weeks old) as most common,^2^, ^8^ time from injury to diagnosis was available in only seven cases in our study, with a median of 8 weeks. Although limited, this subset suggests a tendency toward more chronic presentation in this study. Limb, bone, or the fracture status (open vs. closed) affected did not appear to influence outcome; however, this may reflect the limited number of cases in each group rather than a true lack of association.

The decision to perform plate fixation (PF) for proximal SMCT fractures remains nuanced. While PF is commonly recommended, particularly for closed fractures, some authors advocate conservative management for open and/or comminuted.^2^, ^8^ Studies by Sherlock et al. and Jackson et al. suggest that conservative therapy for proximal SMCT fractures may yield comparable outcomes in survival, return‐to‐work, and convalescence.^2^, ^8^ However, these studies were based on small cohorts (5–7 horses) with limited follow‐up. Jackson et al. reported a 60% return‐to‐work following PF of two open and three closed proximal SMCT fractures, but found no clear association between treatment type (conservative vs. surgical) and long‐term outcome. Their study included fractures at various locations and multiple surgical techniques, complicating interpretation. Notably, they reported a 91.7% return‐to‐work for horses with open proximal SMCT fractures treated conservatively, albeit with longer recovery times. Similarly, Sherlock et al. examined a highly specific subset: open, comminuted fractures of MT4. In that cohort, they reported a 71% return‐to‐work following conservative management, with an average convalescence period of 6 months. Together, these findings support conservative management for open proximal SMCT fractures; however, due to the limited number of cases and the small number of horses treated with PF, they offer little insight into PF outcomes.

In our study, we observed a 76.7% return‐to‐work, with non‐racehorses returning to ridden work in approximately 6 months and racehorses returning to competition after an average of 10 months. Although our racehorses had a more prolonged convalescence, the return‐to‐work rate was comparable to those reported with conservative management, with similar outcomes overall.^2^, ^8^ There was no difference in the proportion of racehorses returning to work compared to non‐racehorses; however, this should be interpreted cautiously given the small sample sizes. It remains possible that other explanatory variables not captured in this retrospective study may have influenced the outcomes.

Based on the outcomes observed in our study, PF may provide benefits in cases of displaced comminuted articular SMCT, particularly in MC2, by offering increased axial support and stability. However, given the small sample size and the absence of a comparison cohort, further studies with larger cohorts and a more balanced treatment approach are needed before definitive recommendations can be made.

Maintaining the independent mobility of the SMCT relative to MC3/MT3 is preferred for successful outcomes, and engaging the palmar/plantar cortex of MC3/MT3 should be reserved for specific scenarios,^1^, ^5^, ^12^ such as when there is insufficient bone for purchase proximally or when fusion of SMCT to MC3/MT3 is intended. However, engaging the cortex may increase the risk of iatrogenic complications, including stress riser formation or propagation of an undetected MC3/MT3 fracture. In this study, one MT3 fracture was identified preoperatively (Horse 25), and that case was managed with independent screw fixation of MT3 and PF of MT4 rather than the MT4 plate screws engaging the plantar MT3 cortex.

A catastrophic MT3 fracture occurred during anesthesia recovery after proximal SMCT fracture repair in Horse 27. Possible contributing factors include displacement of an undetected, non‐displaced MT3 fracture or stress riser formation from drill tract engagement of the plantar cortex (Figure 4).^13^ Similar catastrophic MC3/MT3 fractures have been reported, including one following distal ostectomy.^2^, ^8^ A strategy to reduce this risk is advanced preoperative imaging (e.g., CT), particularly in cases involving high‐energy trauma. In situations where anesthesia or recovery pose increased fracture risk and advanced imaging is unavailable, alternative approaches such as delayed surgery with repeat radiographs after 7–10 days, assisted recovery (e.g., sling/ pool systems), or standing internal fixation should be considered.^13^ Although published reports on standing internal fixation of splint bone fractures are lacking, standing surgical management of splint bones has been described, and retrospective studies on internal fixation of other distal limb fractures, such as P1 and MC3/MT3, support the feasibility of standing repair.^14^, ^15^ These findings suggest that standing plate fixation may be a viable option in select splint bone fracture cases, particularly when minimizing anesthesia‐related recovery risks is a priority.

Surgical site infection occurred in 2/27 cases (7.4%), which is lower than previously reported rates (14.3%–63.6%) likely due to fewer open fractures (5/27, 18.5%) in our study in comparison to others (43%–100%).^2^, ^5^, ^8^ The two which developed a surgical site infection in our study were both open fractures. Given the high success rate of conservative management for displaced, open, and infected fractures of MT4, these cases may be better suited to non‐surgical treatment rather than plate fixation.^2^, ^8^ For displaced and/or articular open fractures, the risks of infection should be weighed against potential displacement and delayed healing, with adequate healing possible if stability is maintained.^1^, ^5^

This study is the first to report on the use of locking compression plates (LCPs), limited contact dynamic compression plates (LC‐DCPs), and locking reconstruction plates for SMCT fractures. Reconstruction plates offer malleability for bone contouring but are weaker due to their annealed soft steel composition.^5^, ^17^ LCPs provide a stable, fixed‐angle construct and facilitate a less invasive approach minimizing soft tissue disruption and preventing screw pull‐out.^16^, ^17^ Given that thread stripping was the only intraoperative complication observed, likely related to the smaller size and softer bone quality of SMCTs, we recommend the use of self‐tapping 3.5 mm screws or fasteners to optimize implant purchase. LC‐DCP plates were exclusively used by a single surgeon in racehorses, with colts/stallions accounting for 75% of these cases; however, the sample size was too small to observe any specific trends related to breed or sex. Our analysis found that neither the type of plate used nor the number of holes had a significant effect on the outcome of treatment. This suggests that, within the parameters of this study, the choice of plate type or configuration may not be a determining factor in the clinical success or failure of the procedure.

Plate removal was performed in three horses, two of which had screws engaging the MC3/MT3 bones. Plate removal is indicated in cases of infection or implant loosening.^1^, ^2^ It is also recommended 3–4 months postoperatively, especially in cases in which MC3/MT3 is engaged, due to the potential for altered biomechanics and pain during strenuous exercise or cold weather.^1^ In cases of persistent lameness with no apparent cause, plate removal may be considered as a diagnostic tool.

Limitations of this study include a small sample size, with few cases in each type of bone affected, which limits the ability to draw strong conclusions. The dataset is highly variable, influenced by surgeon and technique variability, and a heterogeneous population of horses. Additionally, incomplete medical records hinder a more comprehensive analysis, and the retrospective nature of the study introduces potential biases. No factors were found to contribute significantly to outcomes, further emphasizing the need for larger, prospective studies with standardized protocols. These would allow for better comparisons between plate fixation and conservative management for specific fracture types and provide more reliable and generalized insights.

This study reports a 76% success rate for SCMT fractures treated with PF, supporting its use for proximal SMCT fractures. While not evaluated in the present study, PF is generally considered to offer mechanical advantages in terms of axial support and fracture alignment in appropriately selected cases. However, outcomes remain highly dependent on case selection, including fracture configuration, stability, and the presence or absence of infection. PF enhances axial support, fracture alignment, and can reduce complications but success is dependent on careful case selection, considering fracture type, stability, and presence of infection. None of the explanatory variables were significantly associated with return to use, likely due to the small and variable dataset, suggesting that recovery factors are multifactorial and warrant further investigation. Observed complications underscore the importance of precise surgical techniques to optimize outcomes and minimize risks.

## AUTHOR CONTRIBUTIONS

Melly V, MVB: Identified relevant medical records, recorded demographics, compiled, interpreted data, reviewed radiographs, and drafted the manuscript. García‐López JM, VMD, DACVS (Large Animal), DACVSMR: Designed the study, reviewed radiographs, managed six surgical cases, provided data, and edited the manuscript. Stewart HL, VMD, PhD, DACVS (Large Animal): Analyzed data for statistical significance and edited the manuscript. Ortved KF, DVM, PhD, ACVS, DACVSMR: Managed five surgical cases and provided data. Hogan PM, VMD, DACVS (Large Animal): Managed eight cases and provided data. Richardson DW, DVM, DACVS (Large Animal): Managed 10 cases and provided data. Bubeck K, DACVS (Large Animal), DACVSMR: Managed three cases and provided data. All authors reviewed and approved the final manuscript, acknowledging their contributions and ensuring its integrity.

## CONFLICT OF INTEREST STATEMENT

The authors declare no conflicts of interest related to this report.

## Data Availability

The data supporting this study's findings are available from the corresponding author upon reasonable request.
